# Substance use disorder treatment cost-sharing in the 2025 Affordable Care Act Individual Marketplace

**DOI:** 10.1093/haschl/qxag088

**Published:** 2026-04-16

**Authors:** Olivia M Hinds, Skylar B Gross, Christina M Andrews, David M Anderson

**Affiliations:** Department of Health Services Policy and Management, Arnold School of Public Health, University of South Carolina, Columbia, SC 29208, United States; Department of Health Services Policy and Management, Arnold School of Public Health, University of South Carolina, Columbia, SC 29208, United States; Department of Health Services Policy and Management, Arnold School of Public Health, University of South Carolina, Columbia, SC 29208, United States; Department of Health Services Policy and Management, Arnold School of Public Health, University of South Carolina, Columbia, SC 29208, United States

**Keywords:** substance use disorder, ACA Individual Marketplace, cost-sharing, copayments, coinsurance

## Introduction

The Affordable Care Act (ACA) mandates substance use disorder (SUD) treatment as an Essential Health Benefit for ACA Marketplace plans. Although mandated coverage is an important milestone for improving SUD treatment access, SUD benefits are subject to cost-sharing, which may act as a barrier to care.^1^ Moreover, while over 24 million adults were enrolled in an Individual Marketplace plan in 2025,^2^ little is known about SUD cost-sharing requirements for ACA Individual Marketplace enrollees and whether there are differences in these requirements among plan categories. To address this gap, this cross-sectional study examined SUD treatment cost-sharing requirements across plan types in the 2025 ACA Individual Marketplace.

## Methods

We used the 2025 HIX Compare Individual Marketplace dataset for all 504 rating areas.^3^ We collected copayment and coinsurance requirements for inpatient and outpatient SUD treatment. We categorized plans in 4 ways: plan metal level (ie, Bronze [a combination of Bronze and expanded Bronze], Silver, Gold, and Platinum); Silver plan cost-sharing reduction (CSR) level, which reduces cost-sharing for lower income enrollees; on-exchange and off-exchange; and the benchmark on-exchange Silver plan that determines subsidy value.

We calculated the mean and median SUD treatment cost-sharing requirements across each plan category and used chi-square and Wilcoxon signed-rank tests to compare the proportion and median, respectively, across plan categories. Analyses were completed using Stata 19 with a significance level of less than .05. As a robustness check, we compared the mean cost-sharing requirements for SUD treatment with the following inpatient and outpatient treatment groups: (1) medical facilities and (2) mental health (Appendixes S1 and S2).

## Results

Among the 504 Individual Marketplace rating areas operating in 2025, SUD cost-sharing requirements varied substantially by plan type. Across all plan categories, copayment requirements were higher among inpatient than outpatient SUD treatment benefits (Table 1). Across metal levels, Bronze plans had the highest median inpatient per-stay copayment of $500 (IQR: $0–$3000) compared with the lowest in Platinum plans with a median of $350 (IQR: $350–$500) (*P* < .001). The median outpatient copayments ranged from $15 (IQR: $10–$15) for Platinum plans to $50 (IQR: $1–$55) for Bronze plans (*P* < .001). Across CSR levels, inpatient and outpatient copayments decreased as the CSR actuarial value increased. Off-exchange plans had lower median copayments for inpatient SUD ($300; IQR: $0–$800) compared with on-exchange plans ($450; IQR: $0–$950) (*P* < .001), but higher median outpatient SUD treatment copayments ($25; IQR: $5–$40) compared with on-exchange plans ($20; IQR: $0–$40) (*P* < .001).

**Table 1.** SUD cost-sharing requirements for ACA Individual Marketplace plans, 2025.

	Metal levels				Silver CSR levels				Exchange type		Benchmark
	Bronze	Silver	Gold	Platinum	Base	CSR-04	CSR-05	CSR-06	Off	On	Benchmark
14-Digit plan ID rating, dyad count, No.	14 131	55 767	13 627	1509	18 971	12 223	12 221	12 223	13 755	72 395	504
Copay, $											
Mean inpatient SUD***,^a^	1221.81	576.99	631.84	377.16	799.54	850.81	362.14	192.18	499.79	718.82	454.49
Median (IQR) inpatient SUD***	500 (0-3000)	400 (0-800)	500 (250-1000)	350 (350-500)	550 (0-1000)	500 (250-1500)	250 (0-500)	150 (0-300)	300 (0-800)	450 (0-950)	500 (0-550)
Mean outpatient SUD***	41.71	21.08	22.42	12.25	32.23	30.68	13.75	2.17	27.37	23.33	34.58
Median (IQR) outpatient SUD***	50 (1-55)	20 (0-40)	25 (15-30)	15 (10-15)	35 (25-40)	35 (20-40)	15 (5-20)	0 (0-3)	25 (5-40)	20 (0-40)	40 (30-40)
Coinsurance, %											
Mean inpatient SUD***	44.16	33.62	25.92	12.56	37.75	39.21	31.91	23.33	31.94	34.14	39.77
Median (IQR) inpatient SUD***	50 (40-50)	30 (25-40)	20 (20-30)	10 (10-15)	40 (30-50)	40 (40 -40)	30 (30-40)	25 (15-25)	30 (20-40)	35 (25-40)	40 (40-50)
Mean outpatient SUD***	36.05	27.72	24.11	17.58	30.31	30.66	26.56	20.78	28.57	30.41	33.59
Median (IQR) outpatient SUD***^,b^	40 (25-50)	25 (20-40)	20 (20-30)	20 (15-20)	30 (20-40)	30 (20-40)	25 (20-35)	20 (10-30)	20 (20-35)	30 (20-40)	35 (25-40)

Abbreviations: ACA, Affordable Care Act; CSR, cost-sharing reduction; SUD, substance use disorder.

We used the 2025 HIX Compare Individual Marketplace to collect cost-sharing requirements (copayments and coinsurance) for inpatient and outpatient SUD treatment for all 504 rating areas in the United States in 2025. Off-exchange plans were identified as such if the plan was not offered on-exchange. We identified the benchmark plan as the second lowest premium on-exchange Silver plan in each rating area. We may misestimate the benchmark due to partial rating area offerings. Differences in proportions were evaluated by chi-square tests and differences in medians were assessed by the Wilcoxon signed-rank tests. Catastrophic plans were excluded as these plans had miniscule market share in the study period and have substantially different risk adjustment and pricing rules relative to metal-level plans. ***P < .001.

^a^Silver base-01 and Silver CSR-04 were not statistically different from each other (*P* = .721) but were statistically different from the other CSR levels.

^b^Silver base-01 and Silver CSR-04 were not statistically different from each other (*P* = .118) but were statistically different from the other CSR levels.

Coinsurance requirements, which are the percentage of treatment costs for which enrollees must pay once their deductible has been met, were greatest among inpatient SUD treatment services for all plan categories. Median inpatient coinsurance for Bronze plans at 50% (IQR: 40%–50%) was higher than median coinsurance for Platinum plans at 10% (IQR: 10%–15%) (*P* < .001). Similarly, median outpatient coinsurance for Bronze plans at 40% (IQR: 25%–50%) was higher than Platinum plans at 20% (IQR: 15%–20%) (*P* < .001). All Silver-01 base plans compared with CSR-06 had higher median inpatient coinsurance (40% [IQR: 30%–50%] vs 25% [IQR: 15%–25%]; *P* < .001) and median outpatient coinsurance (30% [IQR: 20%–40%] vs 20% [IQR: 10%–30%]; *P* < .001). On-exchange median inpatient and outpatient coinsurance was higher than off-exchange plans (*P* < .001).

When compared with medical facility benefits and mental health treatment benefits, we found similar cost-sharing trends across plans, but at different magnitudes. While the mean inpatient SUD treatment copayments were similar to inpatient facilities, inpatient mental health copayments were substantially lower. For example, Bronze plans had a mean inpatient mental health copayment of $180.67, compared with $1221.81 for SUD treatment and $1229.61 for medical facilities (Appendixes S1 and S2).

## Discussion

Substance use disorder treatment cost-sharing requirements in the 2025 ACA Individual Marketplace vary dramatically across multiple dimensions. Cost-sharing requirements remain greatest, and vary most substantially, across all plan categories for inpatient SUD treatment. As integrated care models have continued to emerge as approaches to improve inpatient-based care for individuals with SUD, it is possible that high cost-sharing requirements may limit inpatient SUD treatment ability to serve as a touchpoint for individuals to engage in SUD treatment.^4^ Our findings suggest that cost-sharing for inpatient SUD treatment mirrors that of medical facility benefits, likely resulting from the Mental Health Parity and Addiction Equity Act,^5^ which prohibits more restrictive cost-sharing for behavioral health treatment; however, with lower cost-sharing for inpatient mental health treatment, enrollees with co-occurring mental health and SUD, which are often treated separately, may delay SUD treatment in exchange for less costly mental health treatment.

Our findings also highlight that copayments and coinsurance for SUD treatment benefits were greatest among Bronze and Silver plans, which are the plans with the greatest enrollment among ACA plan metal levels. These findings highlight particularly high costs required to access SUD treatment, which may result in millions of individuals delaying or forgoing care altogether.

We acknowledge several limitations. First, this study uses a cross-sectional design; however, it is the first study to our knowledge to examine SUD cost-sharing requirements among ACA Individual Marketplace plans. Second, this study does not observe enrollee purchasing decisions; however, a strength of this dataset is that it provides important insight around cost-sharing requirements in off-exchange plans, which are often not included in other publicly available datasets for cost-sharing in Marketplace plans. Third, there is substantial cost-sharing variation across states (Appendixes S3–S6), steering influence on what plan an individual selects to purchase; to capture such variation, we included the benchmark plan as it is one of the most commonly purchased plans for each rating area.^6^ Last, we did not observe utilization of these benefits.

## Conclusion

Cost-sharing requirements for SUD treatment benefits remain high among ACA Individual Marketplace plans and are most salient for inpatient SUD treatment benefits among Bronze and Silver plans. High cost-sharing requirements for SUD treatment benefits may reduce access to care. Future research is needed to examine the impact of SUD cost-sharing requirements on health outcomes.

## Supplementary Material

qxag088_Supplementary_Data
