# Supplementary material for: Substance use disorder treatment cost-sharing in the 2025 Affordable Care Act Individual Marketplace
Source: Health Aff Sch. 2026 Apr 16;4(5):qxag088. doi: 10.1093/haschl/qxag088 (PMC13178459; doi:10.1093/haschl/qxag088)
Supplement: qxag088_Supplementary_Data [file qxag088_supplementary_data.zip › Supplemental Material_Appendices.docx]

Appendix 1: Mean Inpatient and Outpatient Facility Treatment Benefit Cost-Sharing Requirements, 2025

|  |  | Metal Levels | | | | Silver CSR-Levels | | | | Exchange Type | | Benchmark |
| --- | --- | --- | --- | --- | --- | --- | --- | --- | --- | --- | --- | --- |
|  |  | Bronze | Silver | Gold | Platinum | Base | CSR-04 | CSR-05 | CSR-06 | Off | On | Benchmark |
| Copay ($) | Mean Inpatient Facility | 1229.61 | 617.2 | 655.03 | 388.93 | 841.53 | 914.36 | 390.92 | 207.23 | 507.96 | 755.63 | 500.6 |
|  | Mean Outpatient Facility | 440.5 | 288.26 | 278.39 | 171.55 | 403.54 | 428.51 | 169.34 | 74.39 | 237.35 | 311.72 | 227.65 |
| Coinsurance  (%) | Mean Inpatient Facility | 44.16 | 33.68 | 25.88 | 12.56 | 37.89 | 39.2 | 32.04 | 23.33 | 31.94 | 34.17 | 39.82 |
|  | Mean Outpatient Facility | 44.36 | 33.5 | 25.51 | 12.45 | 38.29 | 39 | 31.22 | 23.14 | 32.11 | 34.1 | 39.9 |

Appendix 2: Mean Inpatient and Outpatient Mental Health (MH) Treatment Benefit Cost-Sharing Requirements, 2025

|  |  | Metal Levels | | | | Silver CSR-Levels | | | | Exchange Type | | Benchmark |
| --- | --- | --- | --- | --- | --- | --- | --- | --- | --- | --- | --- | --- |
|  |  | Bronze | Silver | Gold | Platinum | Base | CSR-04 | CSR-05 | CSR-06 | Off | On | Benchmark |
| Copay ($) | Mean Inpatient Facility | 180.67 | 171.2 | 210.94 | 70.1 | 244.87 | 229.87 | 112.84 | 57.02 | 199.05 | 162.41 | 200.78 |
|  | Mean Outpatient Facility | 41.66 | 21.13 | 22.42 | 12.25 | 32.38 | 30.7 | 13.76 | 2.17 | 27.57 | 23.32 | 34.57 |
| Coinsurance  (%) | Mean Inpatient Facility | 44.64 | 33.14 | 25.47 | 14.83 | 37.83 | 38.59 | 31.08 | 22.6 | 32.68 | 33.98 | 39.97 |
|  | Mean Outpatient Facility | 36.05 | 27.73 | 24.11 | 17.58 | 30.31 | 30.66 | 26.56 | 20.78 | 28.59 | 30.41 | 33.59 |

Appendix 3: State-Level Variation in Inpatient Substance Use Disorder Treatment Benefit Copayments

|  | **Inpatient Copay ($)** | | | | | | | |
| --- | --- | --- | --- | --- | --- | --- | --- | --- |
|  | **Bronze** | | **Silver** | | **Gold** | | **Platinum** | |
| **State** | **Mean** | **Std. Dev.** | **Mean** | **Std. Dev.** | **Mean** | **Std. Dev.** | **Mean** | **Std. Dev.** |
| AK | — | — | — | — | — | — | — | — |
| AL | 1700 | 1499.15 | 1625 | 897.36 | 836.84 | 800.82 | — | — |
| AR | 1500 | 1527.53 | 495.97 | 365.88 | — | — | — | — |
| AZ | 1090.91 | 1347.1 | 931.47 | 975.73 | 840.91 | 737.47 | — | — |
| CA | 0 | 0 | 300 | 273.86 | 390.95 | 92.92 | 222.75 | 12.86 |
| CO | 625 | 1188.8 | 1962.69 | 1171.74 | 2000 | 0 | — | — |
| CT | 416.67 | 188.31 | 285.42 | 217.03 | 500 | 0 | — | — |
| DC | — | — | — | — | 550 | 122.47 | 250 | 0 |
| DE | 0 | 0 | 718.75 | 482.34 | 590 | 126.98 | 333.33 | 14.43 |
| FL | 2257.55 | 1170.42 | 479.62 | 699.27 | 579.91 | 416.08 | 320.88 | 95.42 |
| GA | 618.67 | 1000.55 | 405.27 | 551.35 | 567.01 | 518.56 | 350 | 0 |
| HI | 0 | — | — | — | — | — | 350 | 0 |
| IA | 2222.22 | 1339.73 | 1625 | 1030.78 | 2000 | — | — | — |
| ID | 0 | 0 | 793.75 | 441.83 | — | — | — | — |
| IL | 1334.78 | 894.23 | 881.34 | 865.52 | 1080 | 513.46 | — | — |
| IN | 664.38 | 1065.3 | 437.41 | 617.77 | 743.12 | 363.97 | 350 | 0 |
| KS | 2172.41 | 1251.5 | 702.7 | 1000.86 | 1212.12 | 780.93 | — | — |
| KY | 0 | 0 | 220.45 | 213.2 | 400 | 202.55 | — | — |
| LA | 2053.85 | 1042.2 | 1271.88 | 729.45 | 1300 | 509.32 | — | — |
| MA | 1252.21 | 395.98 | 660.79 | 374.71 | 380.46 | 230.34 | 425 | 135.49 |
| MD | 500 | 1031.42 | 718.42 | 725.09 | 560 | 222.8 | 350 | 0 |
| ME | 0 | 0 | 0 | 0 | — | — | — | — |
| MI | 347.49 | 961.92 | 108.82 | 187.73 | 362.32 | 580.85 | — | — |
| MN | 0 | 0 | 263.46 | 544.3 | 250 | 769.48 | — | — |
| MO | 2107.14 | 1151.22 | 886.36 | 1046.7 | 2000 | 0 | — | — |
| MS | 1250 | 1365.39 | 1416.67 | 849.61 | 2000 | 0 | — | — |
| MT | 373.17 | 427.07 | 289.22 | 376.07 | 625 | 233.49 | — | — |
| NC | 2305.88 | 1096.79 | 573.04 | 819.53 | 930.56 | 469.53 | — | — |
| ND | 0 | 0 | — | — | — | — | — | — |
| NE | 1935.48 | 1459.12 | 1625 | 906.96 | 2000 | 0 | — | — |
| NH | 857.14 | 1463.85 | 504.35 | 777.07 | 0 | — | — | — |
| NJ | 333.33 | 288.68 | 350 | 167.08 | 500 | — | — | — |
| NM | 0 | 0 | 0 | 0 | 0 | 0 | — | — |
| NV | 158.82 | 355.63 | 424.86 | 462.85 | 555.56 | 527.05 | — | — |
| NY | 1097.02 | 665.89 | 877.67 | 633.43 | 917.23 | 210.23 | 509.03 | 94.23 |
| OH | 766.53 | 1227.38 | 571.36 | 753.99 | 749.26 | 436.99 | — | — |
| OK | 1271.43 | 1310.98 | 238.41 | 165.86 | 560 | 492.47 | — | — |
| OR | 0 | 0 | — | — | — | — | — | — |
| PA | 426.1 | 997.98 | 712.07 | 863 | 581.72 | 477.87 | 325 | 0 |
| RI | 0 | 0 | — | — | 150 | 173.21 | 0 | 0 |
| SC | 1289.11 | 1338.31 | 421.58 | 765.28 | 304.15 | 719.85 | — | — |
| SD | 0 | 0 | 0 | 0 | — | — | — | — |
| TN | 2046.43 | 1368.74 | 669.12 | 991.04 | 933.33 | 1032.8 | 350 | — |
| TX | 908.7 | 877.67 | 745.35 | 600.09 | 857.39 | 395.5 | — | — |
| UT | 2030.56 | 1308.47 | — | — | 1000 | 0 | 350 | 0 |
| VA | 2000 | 1131.37 | 1537.5 | 956.4 | 1058.33 | 1066.18 | 350 | 0 |
| VT | 0 | 0 | 318.18 | 641.71 | 250 | 433.01 | — | — |
| WA | 870.97 | 1384.24 | 559.44 | 483.21 | 598.39 | 258.62 | — | — |
| WI | 1167.7 | 1262.77 | 522.06 | 569.24 | 1628.91 | 931.15 | 350 | — |
| WV | 0 | 0 | 402.86 | 259.14 | 553.12 | 99.13 | 350 | 0 |
| WY | 1500 | 1566.7 | — | — | 0 | 0 | — | — |

Appendix 4: State-Level Variation in Outpatient Substance Use Disorder Treatment Benefit Copayments

|  | **Outpatient Copay ($)** | | | | | | | |
| --- | --- | --- | --- | --- | --- | --- | --- | --- |
|  | **Bronze** | | **Silver** | | **Gold** | | **Platinum** | |
| **State** | **Mean** | **Std. Dev.** | **Mean** | **Std. Dev.** | **Mean** | **Std. Dev.** | **Mean** | **Std. Dev.** |
| AK | 45 | 26.37 | 25 | 20.42 | 37 | 21.61 | — | — |
| AL | 48.22 | 20.97 | 32.81 | 22.13 | 29.58 | 14.14 | — | — |
| AR | 46.79 | 14.79 | 22.56 | 19.18 | 24.62 | 10.33 | — | — |
| AZ | 31.37 | 29.17 | 18.49 | 18.54 | 15.32 | 12.85 | — | — |
| CA | 24.91 | 29.62 | 32.47 | 18.51 | 34.57 | 1.4 | 14.82 | 1.33 |
| CO | 11.55 | 28.49 | 16.12 | 19.43 | 10.71 | 9.87 | — | — |
| CT | 48.75 | 27.8 | 31.76 | 16.95 | 25 | 8.8 | — | — |
| DC | 50 | 10 | 21.25 | 16.45 | 21 | 7.75 | 20 | 0 |
| DE | 42.14 | 24.55 | 26.54 | 20.55 | 22.33 | 6.51 | 3.33 | 5.77 |
| FL | 58.2 | 23.81 | 21.37 | 23.15 | 33.19 | 20.02 | 11.39 | 3.06 |
| GA | 44.23 | 32.91 | 24.21 | 19.58 | 20.69 | 11 | 5.65 | 5.01 |
| HI | 52.5 | 19.9 | 32.5 | 16.35 | 33.85 | 5.06 | 7.22 | 2.64 |
| IA | 47.46 | 29.15 | 18.69 | 17.44 | 25.42 | 12.63 | — | — |
| ID | 27.89 | 22.43 | 14.62 | 12.33 | 13.86 | 7.28 | 0 | 0 |
| IL | 42.39 | 23.84 | 22.02 | 18.07 | 22.32 | 10.25 | 20 | 0 |
| IN | 41.32 | 24.19 | 22.67 | 15.89 | 17.66 | 9.21 | 10 | 0 |
| KS | 40 | 26.4 | 19.23 | 16.58 | 18.73 | 12.14 | — | — |
| KY | 24.92 | 22.37 | 17.9 | 15.5 | 18.52 | 9.83 | — | — |
| LA | 52.4 | 6.33 | 28.91 | 18.69 | 32.21 | 13.87 | — | — |
| MA | 45.15 | 19.52 | 27.16 | 15.86 | 17.6 | 12.92 | 13.81 | 8.38 |
| MD | 37.65 | 22.8 | 17.41 | 15.41 | 15.26 | 10.39 | 15 | 0 |
| ME | 0.5 | 5 | 0.34 | 3.49 | 0.29 | 2.4 | 0 | 0 |
| MI | 28.36 | 25.23 | 20.63 | 16.85 | 21.96 | 12.32 | — | — |
| MN | 32.02 | 39.96 | 17.77 | 16.38 | 20 | 12.05 | — | — |
| MO | 48.12 | 19.71 | 21.09 | 16.22 | 19.92 | 9.96 | — | — |
| MS | 49.63 | 23 | 25.98 | 17.63 | 25.44 | 10.46 | — | — |
| MT | 24.65 | 24.28 | 21.09 | 15.88 | 22 | 12.2 | — | — |
| NC | 46 | 28.41 | 18.74 | 18.15 | 18.43 | 11.32 | — | — |
| ND | 32.65 | 24.05 | 13.78 | 16.68 | 19.8 | 13.25 | — | — |
| NE | 33.98 | 30.29 | 18.35 | 17.79 | 22.93 | 13.57 | — | — |
| NH | 30.59 | 22.35 | 23.92 | 14.99 | 24.17 | 8.62 | — | — |
| NJ | 53.57 | 26.73 | 34.14 | 23.7 | 25.38 | 19.84 | — | — |
| NM | 0 | 0 | 0 | 0 | 0 | 0 | — | — |
| NV | 17.16 | 20.64 | 14.37 | 17.12 | 20.6 | 14.51 | — | — |
| NY | 34.71 | 23.37 | 20.15 | 12.91 | 17.15 | 11.86 | 13.36 | 4.18 |
| OH | 36.54 | 28.57 | 18.12 | 17.3 | 19.38 | 10.85 | — | — |
| OK | 40.27 | 27.66 | 18.43 | 17.6 | 22.33 | 12.96 | — | — |
| OR | 13.47 | 22.08 | 13.55 | 13.6 | 10 | 7.47 | — | — |
| PA | 36.92 | 35.43 | 31.62 | 25.38 | 21.85 | 13.48 | 0 | 0 |
| RI | 12.5 | 14.43 | 30.43 | 15.78 | 31.88 | 5.12 | 20 | 0 |
| SC | 44.5 | 21.18 | 22.44 | 16.21 | 20.57 | 12.15 | — | — |
| SD | 33.11 | 27.12 | 15.11 | 18.31 | 20.28 | 11.71 | — | — |
| TN | 55.93 | 31.63 | 28.25 | 22.68 | 27.45 | 13.8 | 10 | — |
| TX | 45.19 | 27.49 | 22.55 | 21.27 | 23.99 | 14.14 | — | — |
| UT | 40 | 18.81 | 15.93 | 16.19 | 20.26 | 12 | 5 | 5.14 |
| VA | 45.85 | 28.46 | 20.08 | 18.96 | 22.76 | 10.86 | 10 | 0 |
| VT | 5 | 13.23 | 0 | 0 | 0 | 0 | 0 | 0 |
| WA | 21.23 | 31.99 | 8.41 | 14.96 | 18.93 | 11.72 | — | — |
| WI | 40.73 | 26.69 | 21.04 | 18.29 | 24.96 | 10.07 | 19.39 | 2.42 |
| WV | 40 | 26.13 | 21.82 | 17.51 | 19.58 | 6.92 | 10 | 0 |
| WY | 35.71 | 23.15 | 20.57 | 14.32 | 19.38 | 11.55 | — | — |

Appendix 5: State-Level Variation in Inpatient Substance Use Disorder Treatment Benefit Coinsurance

|  | **Inpatient Coinsurance (%)** | | | | | | | |
| --- | --- | --- | --- | --- | --- | --- | --- | --- |
|  | **Bronze** | | **Silver** | | **Gold** | | **Platinum** | |
| **State** | **Mean** | **Std. Dev.** | **Mean** | **Std. Dev.** | **Mean** | **Std. Dev.** | **Mean** | **Std. Dev.** |
| AK | 39.17 | 8.62 | 32.06 | 4.92 | 28 | 2.54 | — | — |
| AL | 47.42 | 4.4 | 33.28 | 12.95 | 27.09 | 5.13 | — | — |
| AR | 50 | 0 | 35.94 | 9.4 | 26.54 | 4.58 | — | — |
| AZ | 47.3 | 5.59 | 33.46 | 10.37 | 26.7 | 6.95 | — | — |
| CA | 41.22 | 3.28 | 25.1 | 8.09 | 30 | 0 | 10 | 0 |
| CO | 43.95 | 5.8 | 30 | 9.8 | 25.87 | 7.97 | — | — |
| CT | 30 | 11.38 | 34.29 | 10.59 | 13.33 | 7.53 | — | — |
| DC | 32.86 | 12.54 | 17.75 | 8.35 | 32.5 | 5 | — | — |
| DE | 50 | 0 | 36.96 | 11.13 | 24.5 | 3.69 | — | — |
| FL | 43.07 | 14.5 | 38.96 | 10.65 | 27.13 | 7.67 | 10.34 | 1.82 |
| GA | 45.25 | 9.17 | 34.44 | 9.79 | 25.21 | 6.84 | 20 | 0 |
| HI | 44.44 | 5.27 | 29.58 | 8.46 | 27.69 | 3.3 | — | — |
| IA | 49.81 | 1.36 | 33.42 | 11.35 | 25.55 | 4.68 | — | — |
| ID | 37.07 | 15.6 | 31.93 | 12.71 | 17.93 | 6.62 | 10 | 0 |
| IL | 47.73 | 6.06 | 34.48 | 11.15 | 28.32 | 7.95 | 20 | 0 |
| IN | 41.21 | 12.72 | 35.64 | 9.38 | 22.75 | 5.14 | — | — |
| KS | 48.29 | 3.79 | 34.62 | 11.06 | 30.94 | 8.61 | — | — |
| KY | 46.14 | 4.9 | 34.39 | 11.56 | 29.43 | 10.3 | — | — |
| LA | 43.55 | 7.61 | 29.85 | 14.23 | 25.94 | 9.21 | — | — |
| MA | 16.88 | 9.75 | 17.23 | 8.44 | 22.14 | 9.3 | — | — |
| MD | 41.11 | 5.75 | 32.24 | 13.36 | 31.82 | 7.24 | — | — |
| ME | 47.22 | 6.6 | 24.84 | 7.18 | 30.29 | 2.4 | 20 | 0 |
| MI | 48.59 | 3.49 | 27.54 | 11.06 | 25.44 | 4.39 | — | — |
| MN | 40.71 | 13.57 | 22.77 | 11.25 | 21.06 | 5.8 | 19.21 | 3.44 |
| MO | 42.79 | 11.2 | 33.56 | 10.21 | 23.39 | 5.26 | — | — |
| MS | 43.98 | 9.99 | 32.03 | 11.38 | 22.92 | 4.35 | — | — |
| MT | 50 | 0 | 31.42 | 6.85 | 24.38 | 5.92 | — | — |
| NC | 48.93 | 3.09 | 36.81 | 10.93 | 26.31 | 6.78 | — | — |
| ND | 50 | 0 | 31.81 | 11.44 | 25.93 | 5.91 | — | — |
| NE | 46.52 | 9.18 | 32.95 | 10.52 | 26.55 | 4.06 | — | — |
| NH | 40.94 | 7.79 | 28.8 | 10.08 | 25.59 | 7.88 | — | — |
| NJ | 43.33 | 9.85 | 28.74 | 14.21 | 26.25 | 9.8 | — | — |
| NM | — | — | — | — | — | — | — | — |
| NV | 46.74 | 5.19 | 36.43 | 11.3 | 27.41 | 6.3 | — | — |
| NY | 45.85 | 7.37 | 22.07 | 12.12 | 30 | 14.23 | 10 | 0 |
| OH | 45.43 | 8.69 | 32.61 | 9.2 | 26.18 | 6.87 | — | — |
| OK | 49.88 | 2.97 | 34.04 | 10.83 | 27.8 | 6.53 | — | — |
| OR | 34.26 | 13.63 | 25.1 | 11.29 | 21.16 | 5.47 | — | — |
| PA | 47.55 | 5.11 | 34.55 | 9.41 | 24.77 | 7.5 | — | — |
| RI | 15 | 8.66 | 19.36 | 11.45 | 15 | 5.22 | — | — |
| SC | 44.53 | 10.24 | 33.35 | 11.86 | 25.91 | 7.19 | — | — |
| SD | 50 | 0 | 35.14 | 8.02 | 26.94 | 2.99 | — | — |
| TN | 48.67 | 3.41 | 36.98 | 11.38 | 26.65 | 4.45 | — | — |
| TX | 46.95 | 5.66 | 34.63 | 10.06 | 28.48 | 7.8 | — | — |
| UT | 44.58 | 12.63 | 29.95 | 11.83 | 24.06 | 5.16 | 10 | 0 |
| VA | 38.05 | 8.34 | 29.46 | 11.15 | 22.58 | 5.56 | 15 | 0 |
| VT | 50 | 0 | 36.73 | 14.62 | 26.67 | 5.77 | 10 | 0 |
| WA | 36.86 | 11.66 | 26.37 | 9.4 | 22.68 | 8.61 | — | — |
| WI | 46.37 | 8.84 | 32.91 | 11.03 | 26.16 | 6.83 | 20 | 0 |
| WV | 48.33 | 3.74 | 32.5 | 9.13 | 25 | 0 | — | — |
| WY | 48 | 4.14 | 29.89 | 9.24 | 23.89 | 3.2 | — | — |

Appendix 6: State-Level Variation in Outpatient Substance Use Disorder Treatment Benefit Coinsurance

|  | **Outpatient Coinsurance (%)** | | | | | | | |
| --- | --- | --- | --- | --- | --- | --- | --- | --- |
|  | **Bronze** | | **Silver** | | **Gold** | | **Platinum** | |
| **State** | **Mean** | **Std. Dev.** | **Mean** | **Std. Dev.** | **Mean** | **Std. Dev.** | **Mean** | **Std. Dev.** |
| AK | 37.5 | 2.74 | — | — | — | — | — | — |
| AL | 40 | 0 | — | — | — | — | — | — |
| AR | — | — | — | — | — | — | — | — |
| AZ | 42.31 | 10.13 | 32.5 | 2.64 | 20 | 0 | — | — |
| CA | — | — | 30 | 5.07 | — | — | — | — |
| CO | 36.54 | 5.58 | 26.29 | 10.03 | 23.86 | 8.06 | — | — |
| CT | 27.86 | 11.4 | 25 | 5.16 | 10 | 0 | — | — |
| DC | 20 | 0 | — | — | — | — | — | — |
| DE | — | — | — | — | — | — | — | — |
| FL | 30.47 | 18.4 | 27.3 | 12.1 | 31.49 | 5.92 | — | — |
| GA | 39.92 | 12.08 | 29.92 | 9.02 | 16.13 | 4.47 | — | — |
| HI | — | — | — | — | — | — | — | — |
| IA | 40 | 0 | — | — | — | — | — | — |
| ID | 25 | 15.21 | 30 | 8.24 | — | — | — | — |
| IL | 44.29 | 7.68 | 44.26 | 7.98 | 28.13 | 4.03 | — | — |
| IN | 29.34 | 13.55 | 29.35 | 11.77 | 20 | 0 | — | — |
| KS | 43.64 | 4.92 | 37.67 | 2.58 | — | — | — | — |
| KY | 40 | 0 | 15 | 7.97 | — | — | — | — |
| LA | 36.36 | 4.87 | 11.92 | 5.76 | 10 | 0 | — | — |
| MA | — | — | 10 | 0 | 10 | 0 | — | — |
| MD | 42.5 | 4.47 | 25 | 10.16 | 32.5 | 2.67 | — | — |
| ME | 43.92 | 8.69 | 20 | 0 | — | — | — | — |
| MI | 50 | 0 | 35 | 0 | — | — | — | — |
| MN | 30.83 | 17.76 | 19.96 | 10.32 | 15.96 | 6.72 | 19.21 | 3.44 |
| MO | 31.49 | 11.67 | 28.71 | 8.68 | — | — | — | — |
| MS | 34 | 12.21 | 20 | 0 | 20 | 0 | — | — |
| MT | 37.78 | 10.03 | 33.59 | 13.47 | 25.45 | 12.93 | — | — |
| NC | 44.38 | 5.04 | — | — | — | — | — | — |
| ND | — | — | 16 | 5.03 | 10 | 0 | — | — |
| NE | 31.76 | 13.34 | — | — | 28.33 | 7.91 | — | — |
| NH | 36.67 | 2.58 | 20.63 | 6.23 | — | — | — | — |
| NJ | 42.5 | 10.35 | 25.36 | 13.47 | — | — | — | — |
| NM | — | — | — | — | — | — | — | — |
| NV | 41.67 | 6.99 | 33.81 | 12.45 | 27.22 | 2.64 | — | — |
| NY | 49.08 | 4.21 | 15.43 | 5.53 | 20 | 0 | — | — |
| OH | 33.81 | 10.62 | 28.51 | 6.7 | 20 | 0 | — | — |
| OK | 45.71 | 6.9 | 36.99 | 11.78 | 30.93 | 6.8 | — | — |
| OR | 30 | 20.75 | — | — | — | — | — | — |
| PA | 50 | 0 | 35 | 0 | 10.77 | 2.77 | — | — |
| RI | 10.71 | 1.89 | 16.67 | 4.44 | — | — | — | — |
| SC | 25.28 | 8.85 | 20 | 0 | — | — | — | — |
| SD | — | — | 25 | 0 | — | — | — | — |
| TN | 46.5 | 4.89 | 35 | 0 | — | — | — | — |
| TX | 44.72 | 5.32 | 35.76 | 11.54 | 33.32 | 5.68 | — | — |
| UT | 32.67 | 17.71 | 30.69 | 12.3 | 20 | 0 | — | — |
| VA | 33.31 | 5.2 | 23.81 | 9.23 | 20.51 | 5.18 | 15 | 0 |
| VT | 50 | 0 | 21.25 | 12.17 | — | — | — | — |
| WA | 31.35 | 17.23 | 21.88 | 5.12 | — | — | — | — |
| WI | 35.51 | 11.08 | 18.78 | 9.05 | 31.19 | 10.17 | — | — |
| WV | 40 | 0 | — | — | — | — | — | — |
| WY | 45 | 5.48 | 32.5 | 7.58 | 22.5 | 2.74 | — | — |
